# Bone conduction stimulated VEMPs by using the B250 transducer to assess the nerve of origin of sporadic vestibular schwannomas

**DOI:** 10.1038/s41598-024-78060-8

**Published:** 2024-11-03

**Authors:** Torsten Rahne, Stefan K. Plontke, Christian Strauss, Karl-Johan Fredén Jansson, Bo Håkansson, Laura Fröhlich

**Affiliations:** 1 Department of Otorhinolaryngology, Head and Neck Surgery, University Medicine Halle, Ernst-Grube-Str. 40, 06120 Halle (Saale), Germany; 2 Department of Neurosurgery, University Medicine Halle, Halle (Saale), Germany; 3 Department of Electrical Engineering, Chalmers University of Technology, Gothenburg, Sweden; 4 Present Address: Department of Otolaryngology, Head and Neck Surgery, University Hospital Bonn, Bonn, Germany

**Keywords:** cVEMP, oVEMP, Bone conduction, Transducer, Nerve of origin, Threshold, Biomedical engineering, Health care

## Abstract

Vestibular evoked myogenic potentials (VEMPs) are a tool to assess otolith function and a component of sensor specific vestibular diagnostics. The aim of the present study was to measure VEMP trough bone conducted (BC) stimulation using the B250 prototype and to report amplitudes, latencies and threshold levels for patients before resection of a sporadic unilateral vestibular schwannoma (VS) in order to assess function regarding to the reported nerve of origin. Twenty-seven participants (9 male/18 female) with a mean age of 55.9 years (SD: 10.8) were included for the analysis. In the side contralateral to the tumor, in 24 (89%) of the patients cVEMP could be measured, while oVEMP were recordable in 20 patients (74%). For patients with inferior vestibular nerve of origin (n = 11), cVEMP amplitudes of the affected side were significantly lower as compared to the non-affected side, while the force threshold level was increased. No statistically significant differences were observed for neither, oVEMP amplitudes nor threshold levels in the group with superior vestibular nerve of origin (n = 7). Across groups, p13 latency was significantly increased in the affected ear while all other VEMP latencies were not different between the ears. The B250 transducer was applicable to all participants of the clinical cohort. The sample size, however, was too low for a reliable statistical analysis and only allowed for exploratory analysis.

## Introduction

Vestibular function is important for human beings as balance and control of eye movements during head movements are key for an active daily life. Function loss of the vestibular system can induce dizziness, nausea, and falls. The vestibular labyrinth as sensor system for linear and angular acceleration detection consists of the semicircular canals and the otolith organs. A differentiated functional diagnosis of the individual components is essential to identify pathologies and enable customized therapy.

Cochleovestibular schwannomas (usually termed ‘vestibular schwannomas (VS)’ or ‘acoustic neuromas’) are benign tumors of the eight cranial with a lifetime prevalence of more than 1 per 500 persons^1^, most often arising from the superior (SVN) or inferior vestibular nerve (IVN) in the internal auditory canal and/or the cerebellopontine angle^2–4^. Preoperative origin differentiation may influence the decision on treatment. Both, IVN and SVN are afferent pathways that are almost exclusively specific for the vestibular sensors. The utricle is mainly innervated by the SVN, while the IVN mainly innervates the saccule^5–7^. It has been shown that a scoring system based on vestibular testing can predict the nerve of origin with a good correlation to the intraoperative observation^8^.

Vestibular evoked myogenic potentials (VEMPs) as a component of sensor specific vestibular diagnostics are a tool to assess otolith function. Acoustic or vibratory stimulation of the labyrinth evoke vestibular responses. Cervical VEMPs (cVEMPs) reflect saccular function and are recorded from the ipsilateral sternocleidomastoid muscle^9^. Utricular function is assessed with ocular VEMPs (oVEMPs) which are recorded from the contralateral extraocular muscles^10,11^.

With oVEMP stimulation a series of powerful vibratory pulse trains leads to large amplitudes^12–14^. The B250 transducer for BC stimulation is a new, more powerful prototype transducer for both cVEMP and oVEMP stimulation developed in a collaboration between researchers at Chalmers University of Technology in Gothenburg, Sweden and Ortofon A/S in Nakskov, Denmark^15^. Initial data for VEMP stimulation using B250 prototypes showed significantly lower stimulus response thresholds in the hearing level range compared to air conducted (AC) stimulation and thus avoided the risk for exposing patients to hazardous high sound levels^15,16^. Furthermore, the AC stimulation has been shown to result in low response rates (RR), especially in patients with conductive hearing loss^17,18^. Conventionally, the Minishaker B&K4810 has been used for BC stimulated VEMP as audiometric transducers such as the Radioear B71 and B81 (Radioear, New Eagle, USA) generate too low output force at low frequencies as those are intended for audiometry. However, the Minishaker, which is operator dependent and bulky (1.1 kg), requires an additional power amplifier when driven by the diagnostic equipment. The B250 can be driven without a power amplifier as it has a resonance peak at 250 Hz with a force sensitivity of approximately 140 dB relative to 1 µNewton per volt or 27 dB higher than the Radioear devices^19^. It weighs only 80 g and can easily be attached either to the mastoid using a steelspring headband P-3333 or to the forehead using a neoprene headband. The VEMP parameters obtained with B250 when used on the mastoid have shown to be similar to those obtained with the Minishaker on the forehead if the stimulation signal polarity is reversed (rarefaction). In comparison with AC stimulation, B250 is expected to give longer n10 (+ 1.1 ms) and n23 (+ 1.6 ms)^20^.

The aim of the present study was to measure BC stimulated VEMPs by using the B250 prototype and report amplitudes, latencies and threshold levels for patients before resection of a sporadic unilateral VS in order to assess function regarding to the reported nerve of origin.

## Methods

### Patient demographics

We conducted a prospective exploratory study to measure VEMPs in adult patients who were scheduled for VS surgery at a tertiary university medical center in a time period of six months in 2019. The study was performed in accordance with the Declaration of Helsinki and approved by the ethical review board of the Medical Faculty of the Martin Luther University Halle-Wittenberg (approval number 2017-103). Informed consent was obtained from all participants who underwent vestibular testing as part of the routine clinical protocol one day before surgery. Tumor size was specified according to Koos^21^ as reported in the patient’s chart. The hearing function was assessed by measuring pure-tone audiometry and averaging the thresholds at 0.5, 1, 2, and 4 kHz (4PTA_0.5−4 kHz_). Speech perception was measured as German (Freiburg) monosyllabic word recognition score at a sound pressure level of 65 dB (WRS_65_) or above to assess the maximum word recognition score (WRS_max_). Hearing classes according to the AAO-HNS^22^ or Gardner-Robertson (GR)^23^ including German speech audiometry^24^ were determined based on the 4PTA_0.5−4 kHz_ and WRS_max_. The nerve of origin was intraoperatively determined by an experienced neurosurgeon.

### VEMP recordings

VEMP were measured to assess the vestibular function on both, the tumor affected (AS) and the non-affected side (NAS) by using the Eclipse Platform (Interacoustics, Middelfart, Denmark). Vibratory stimulation was applied to the mastoids by using a B250 prototype transducer^15^. Single cycle tone bursts with a frequency of 250 Hz (0 rise/fall time; plateau time 4 ms, rarefaction) were presented with a stimulation rate of 8 Hz and triggered by the internal trigger of the Eclipse system. Stimulation force level was 150 dB relative to 1 µNewton and calibrated according to ISO 389-6:2007-10 as peak-to-peak equivalent vibratory force level (peVFL). Therefore, the signals were delivered to a B250 connected to an artificial mastoid 4930 (Brüel & Kjaer Sound & Vibration Measurement A/S, Kopenhagen, Denmark) with a static force of 5.4 N and output force was recorded with an InfiniiVision 2000 X-Series oscilloscope (Keysight, Santa Rosa, USA).

The B250 has a wider and slightly concave surface compared to the audiometric Radioear devices to more firmly attach to the patient’s head, which has shown to affect the calibration values only for frequencies above 1 kHz where the response becomes flatter^20^. As the pad sensitivity is not affected by the B250 surface below 1 kHz, the same pad sensitivity correction values as for B71 and B81 were used when calibrating the transducer for the 250 Hz tone burst.

Lower levels were additionally applied to measure the VEMP threshold level with a step size of 5 dB. For cVEMP recording, surface electrodes were placed on the upper half of the ipsilateral sternocleidomastoid muscles with a reference electrode on the sternum. The participants were seated in a sound-attenuated booth and were instructed to rotate their heads toward the non-stimulated ear. The electromyogram (EMG) amplitude was continuously recorded, and visual feedback was delivered to the participants for maintaining constant muscle tension^25^. After recording at least 200 artifact free epochs per stimulation level, the epochs were averaged and the first positive–negative peak (p13–n23) of the averaged EMG was defined as the cVEMP. The p13-n23 amplitude was then referenced to the root mean square prestimulus EMG and used for further analysis.

For oVEMP recording, electrodes were placed underneath the participants’ eyes to enable a bipolar recording. EMG was recorded while the participants were asked to look maximally upwards. The first negative–positive peak (n10–p15) of the contralaterally measured averaged EMG was defined as the oVEMP amplitude. For cVEMP and oVEMP, the asymmetry ratios (AR) of amplitudes between the tumor-affected side (AS) and the non-affected side (NAS) was determined using the VEMP amplitudes at a force level of 150 dB by calculating the differences and dividing by the sum of amplitudes^8^. If no VEMP could be detected, amplitudes were set to zero.

### Statistical analysis

The amplitudes, latencies, thresholds and ARs of cVEMP and oVEMP were descriptively analyzed. Participants were allocated according to the intraoperative findings in groups with intraoperatively identified IVN or SVN origin, or no identified origin. Mean and standard deviations (SD) as well as percentiles were calculated accordingly for the groups. Paired two-sided *t*-tests were applied to compare the amplitudes, latencies and thresholds between the affected and non-affected sides (Alpha = 0.05). All statistical analyses were performed using SPSS software version 28 (IBM, Ehningen, Germany). Post hoc power analysis was performed for the amplitude ratios to determine the power or the required sample size.

## Results

### Participants

Twenty-seven participants (9 male/18 female) with a mean age of 55.9 years (SD: 10.8) were included for the analysis. Table 1 shows the demographic data. Tumor origin was IVN in 11 cases, SVN in 7 cases and not determinable in 9 cases.

**Table 1:** Demographic data.

Characteristics	Nerve of tumor origin			
	IVN	SVN	Not determinable	All
Number	11	7	9	27
Age, mean (SD), years	54.27 (9.5)	53.0 (13.4)	60.1 (10.0)	55.9 (10.8)
Median [25th, 75th percentiles]	55 [46,63]	55 [37,58]	56 [51,70]	55.0 [48,65]
Men/women, N	4/7	1/6	4/5	9/18
Affected side, right/left, N	5/6	3/4	5/4	13/14
Koos class, mean (SD), years	3.3 (0.82)	2.1 (0.69)	3.4 (0.79)	3.0 (0.93)
Median [25th, 75th percentiles]	3.5 [2.74,4]	2.0 [2, 3]	4.0 [3, 4]	3 [2, 4]
AAOHNS class, mean (SD), years	2.55 (1.3)	2.43 (1.3)	2.78 (1.2)	2.59 (1.2)
Median [25th, 75th percentiles]	2 [1, 4]	2 [1, 4]	3 [1.5,4]	3 [1, 3]
GR class, mean (SD), years	1.64 (1.6)	2.14 (0.9)	2.89 (1.5)	2.63 (0.50)
Median [25th, 75th percentiles]	2 [1, 5]	2 [1, 3]	3 [1.5,4]	3 [1, 3]
WRS_65_ Affected side, mean (SD), %	42.5 (40.2)	53.6 (39.7)	29.4 (40.5)	41.0 (39.8)
Median [25th, 75th percentiles]	50 [0,85]	65 [0,90]	10 [0,70]	50 [0,85]
WRS_max_ Affected side, mean (SD), %	57.7 (41.9)	70.0 (32.3)	52.8 (41.6)	59.3 (38.4)
Median [25th, 75th percentiles]	75 [0,100]	80 [40,100]	55 [2.5,92.5]	75 [15,100]
PTA_4_ Affected side, mean (SD), dB HL	56.9 (33.9)	49.6 (29,6)	59.4 (28.9)	55.9 (30.3)
Median [25th, 75th percentiles]	50 [29,65]	45 [23,80]	62.5 [35,69]	58.75 [29,68]
PTA_4_ Non-affected side, mean (SD), dB HL	16.1 (8.3)	16.1 (7.9)	20.3 (9.3)	17.5 (8.5)
Median [25th, 75th percentiles]	13.75 [10, 20]	12.5 [10, 21]	18.8 [13, 28]	13.75 [11, 21]

*AAOHNS* American Academy of Otolaryngology, Head and Neck Surgery; *dB HL* decibel hearing level; *GR* Gardner Robertson; *IVN* inferior vestibular nerve; *N* number; *PTA*_*4*_ pure-tone average over 0.5, 1, 2, and 4 kHz; *SD* standard deviation; *SPL* sound pressure level; *SVN* superior vestibular nerve; *WRS*_*65*_ word recognition score at 65 dB SPL; *WRS*_*max*_ maximum word recognition score.

### VEMP

Table 2 shows the amplitudes, latencies, and thresholds for cVEMPs and oVEMPs for the affected and non-affected side for all participants across all tumor origin groups. Considering the side contralateral to the tumor (NAS), in 24 (89%) participants cVEMPs could be measured, while oVEMP were recordable in 20 participants (74%). In two of the three patients without recordable cVEMPs, oVEMPs could be recorded. The response rates were lower if the VS affected side was stimulated. In patients with VS the p13 latency (t(14) = 4.2, *p* < 0.001) and the thresholds (t(14) = 2.6, *p* = 0.021) were significantly increased while the cVEMP amplitude was significantly decreased (t(25) = − 2.2, *p* = 0.040) as compared to the non-affected side. For oVEMP, only the amplitude was significantly decreased (t(25) = − 2.7, *p* = 0.014).

**Table 2:** VEMP responses for all participants.

Characteristics	Non-affected side	Affected side	*p*
cVEMP			
p13 latency, mean (SD), ms	15.5 (2.2)	17.2 (1.9)	** < 0.001**
Median [25th, 75th percentiles]	15.2 [13.7,17.2]	17.5 [16,18.7]	
n23 latency, mean (SD), ms	25.9 (1.9)	26.39 (2.1)	0.074
Median [25th, 75th percentiles]	25.7 [24.8,27.0]	27 [24.3,27.8]	
p13-n23 amplitude^a^, mean (SD)	1.79 (1.83)	0.95 (1.10)	**0.040**
Median [25th, 75th percentiles]	1.54 [0.5,2.0]	0.73 [0.0,1.5]	
Threshold, mean (SD), dB nHL	65 (5.5)	68 (4.9)	**0.021**
Median [25th, 75th percentiles]	65 [60,70]	70 [65,70]	
oVEMP			
n10 latency, mean (SD), ms	11.8 (1.9)	12.3 (1.7)	0.250
Median [25th, 75th percentiles]	11.3 [10.4,14.00]	12.0 [11.3,13.7]	
p15 latency, mean (SD), ms	15.8 (2.1)	16.0 (1.68)	1.000
Median [25th, 75th percentiles]	15.2 [14.3,16.9]	15.3 [14.7,17.7]	
n10-p15 amplitude, mean (SD), µV	4.6 (5.1)	2.2 (3.1)	**0.014**
Median [25th, 75th percentiles]	3.4 [0.0,5.9]	0 [0.0,4.3]	
Threshold, mean (SD), nHL	71 (6.8)	80 (7.9)	0.742
Median [25th, 75th percentiles]	70 [70,75]	65 [65,80]	

^a^normalized to mean eletromyogram amplitude.

Significant values are in bold.

*nHL* normalized hearing level; *SD* standard deviation.

**Fig. 1:**
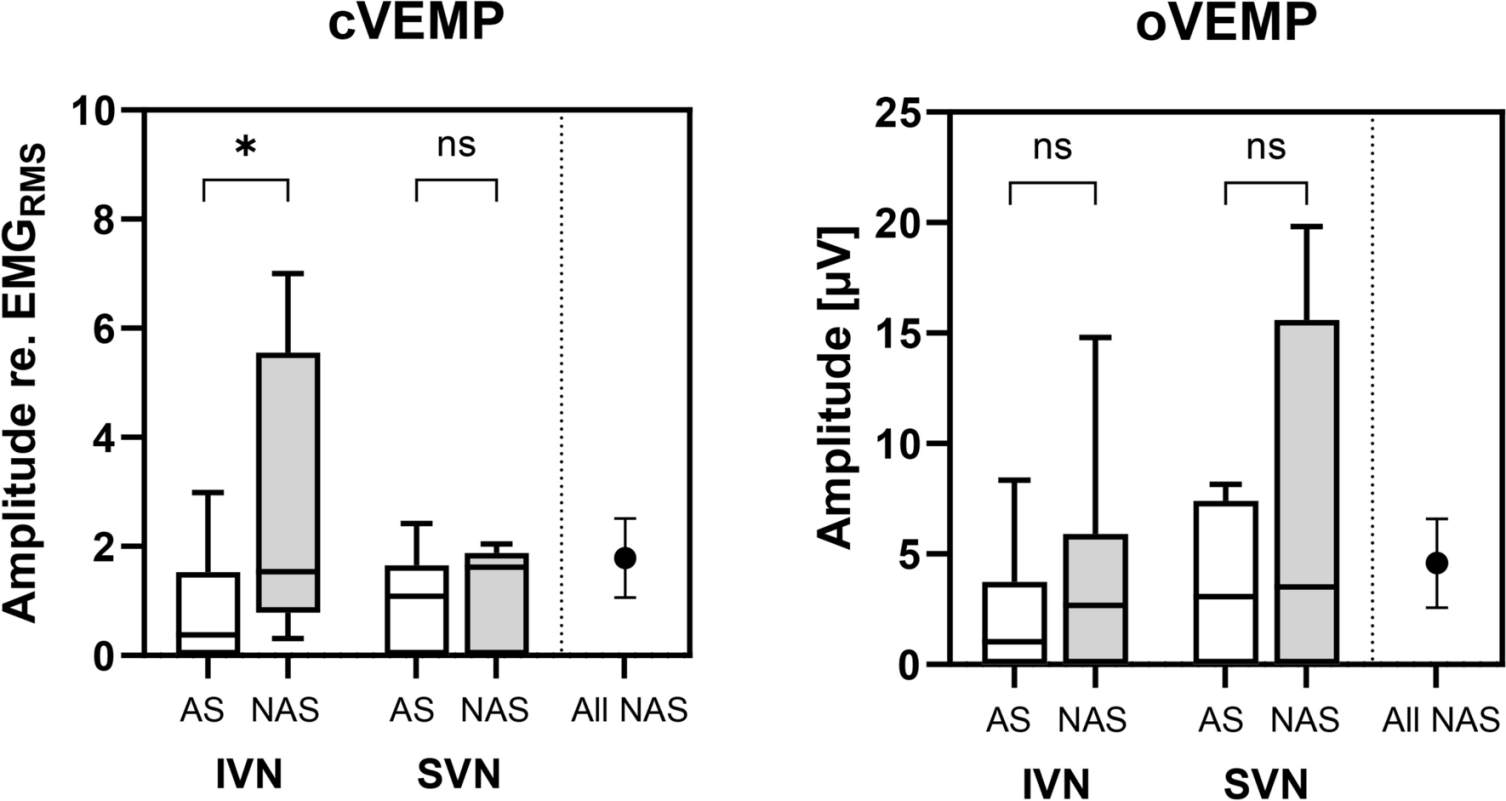
Amplitudes of cVEMP (left, referenced to the root mean square of prestimulus EMG) and oVEMP (right) for the tumor affected (AS, white) and the non-affected sides (NAS, grey). Boxplots show median, 25 and 75 percentiles, and minimum and maximum. The data for IVN (n = 11) and SVN origin (n = 7) are shown in comparison to the mean (+ /− 95% confidence interval) NAS amplitudes of all participants. Significant differences are marked with asterisks (*p < 0.05; *ns* not significant).

**Fig. 2:**
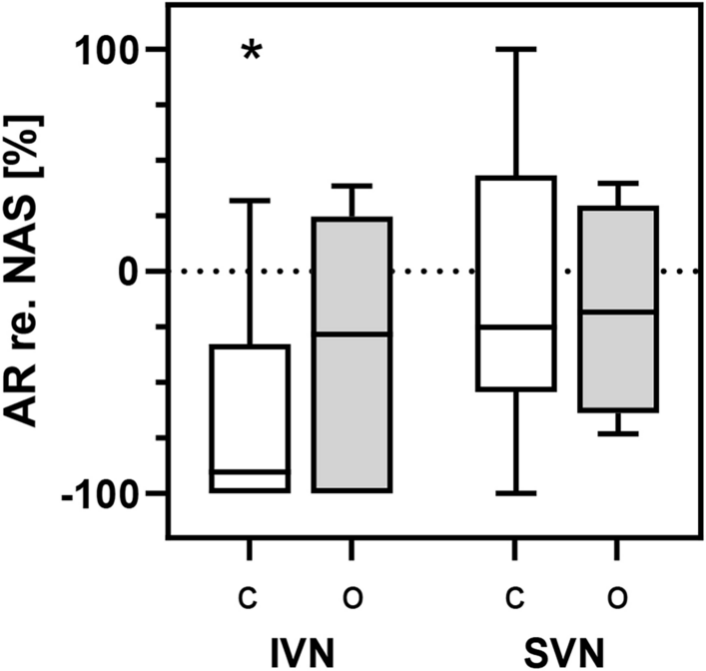
Amplitude asymmetry ratio (AR) for cVEMP (C, white) and oVEMP (O, grey) responses relatively to the non-affected side (NAS). Boxplots show median, 25 and 75 percentiles, and minimum and maximum for participants with identified IVN (n = 11) and SVN origin (n = 7).

Figure 1 shows the amplitudes of cVEMPs and oVEMPs for both, the tumor affected side and the non-affected side, grouped for patients with IVN and SVN tumor origin and those, where the tumor origin could not be determined. For patients with IVN nerve of origin, cVEMP amplitudes of the affected side were significantly lower as compared to the non-affected side (t(9) = 0.03). No statistically significant difference between the affected and non-affected sides of cVEMP and oVEMP was observed for neither, cVEMP nor oVEMP amplitudes in the SVN group. The median cVEMP amplitudes of the non-affected sides were 1.59 (IVN) and 1.62 (SVN) and not significantly different. The ARs for all tumor location groups are shown in Fig. 2. For the IVN group, the AR was − 63.4 (SD: 52.7) for the cVEMP and − 36.0 (SD: 59.39) for the oVEMP. For the SVN group, the AR was − 10.8 (SD: 67.4) and for the cVEMP and − 17.5 (SD: 48.3) for the oVEMP. One-sample t-test showed that only the AR for the cVEMP in the IVN group was significant with a power of 0.94 and an effect size of 1.203. For the observed oVEMP AR in the SVN group, a power analysis using the observed AR with power of 0.80 would result in a required sample size of N = 61.

**Fig. 3:**
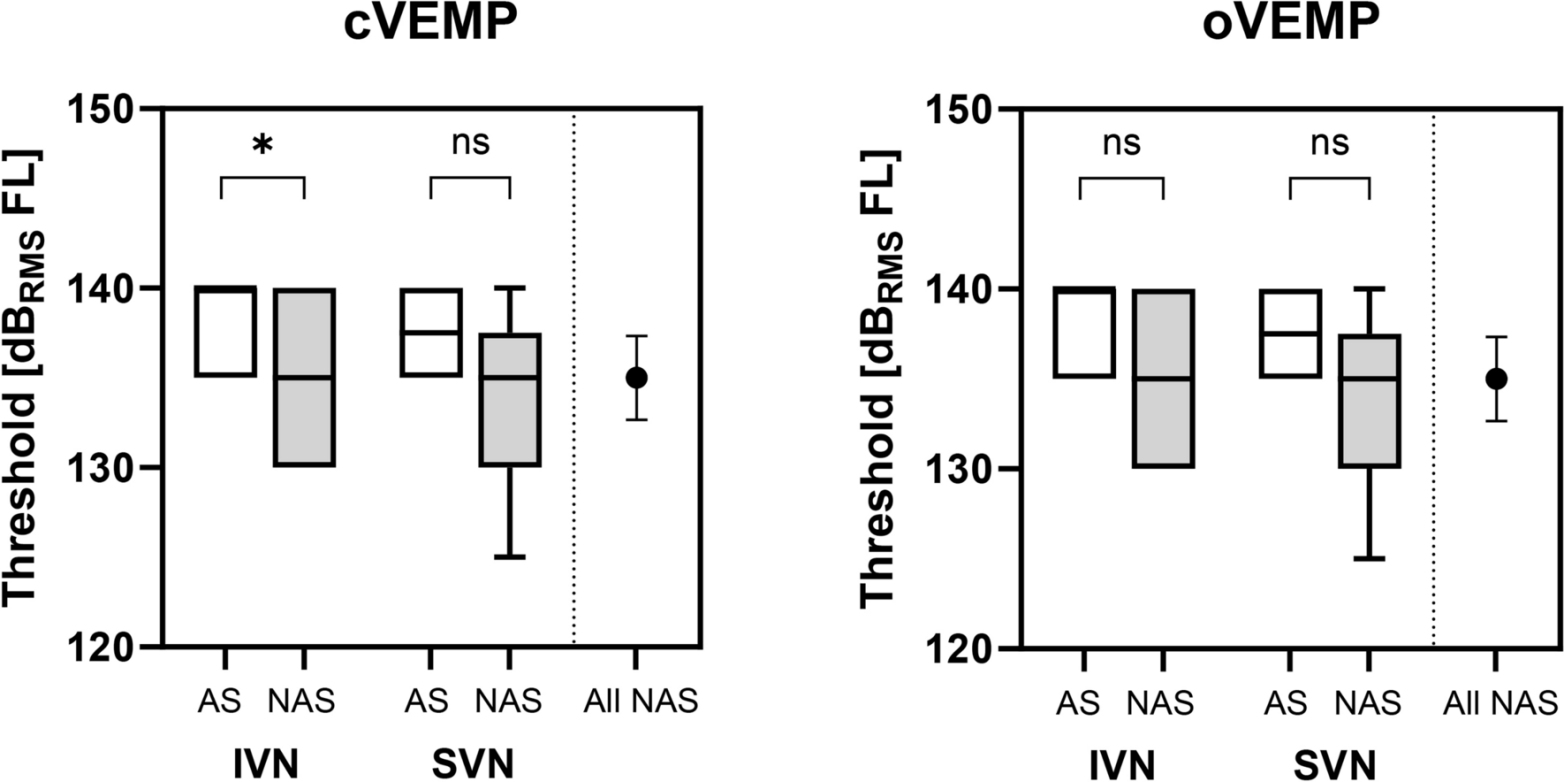
Thresholds for evoking cVEMP (left) and oVEMP responses (right) for the tumor affected (AS, white) and the non-affected side (NAS, grey). Boxplots show median, 25 and 75 percentiles, an minimum and maximum. The data for IVN (n = 11) and SVN origin (n = 7) are shown in comparison to the mean (+ /− 95% confidence interval) NAS amplitudes of all participants. Significant differences are marked with asterisks (*p < 0.05; *ns* not significant).

Threshold stimulation levels for cVEMP and oVEMP are shown in Fig. 3. For patients with IVN nerve of origin, cVEMP thresholds of the affected side were significantly increased as compared to the non-affected side (t(4) = 0.05). No statistically significant difference was observed for cVEMP and oVEMP thresholds.

## Discussion

Cervical and ocular VEMP responses could be evoked in the majority of participants using the B250 transducer. The response rates for both the VS affected and the contralateral, non-affected sides were higher than in a previous study using the B81 clinical routine transducer with lower output force level^14^. Considering the non-affected side, latencies were within the range of reported data using other bone-conduction transducers^10,14,26^. However, we observed a shift of 1–2 ms towards larger latencies which will be investigated in a further study.

The large response rate for cVEMP and oVEMP is attributed to the increased force output of the B250 transducer compared to previously used transducers^20^. Since the VEMP response rate declines with age, using powerful BC stimulation are needed for functional vestibular diagnostic across the entire lifespan. The data of the study provide evidence for the application to a clinical cohort with a typical age distribution.

Only in the IVN but not in the SVN group did the applied cVEMP recordings show significantly reduced amplitudes and increased thresholds in the affected side. This was attributed to a functional deficit of the inferior vestibular nerve that mainly innervates the sacculus. No differences between the affected side and the non-affected sides were observed for oVEMP amplitudes in both groups (IVN, SVN). This supports previous attempts to predict the nerve of tumor origin by a composite score of video head-impulse testing (vHIT) and VEMP with better outcome^8,27^. The sensitivity for predicting tumor origin was lower for oVEMP compared to cVEMP. The sensitivity of oVEMP could potentially be further improved by using forehead stimulation of BC VEMP to reduce inter-side variations in mastoid coupling. As the utricle is innervated by the IVN only, the saccule is also innervated by the SVN via the Voit’s nerve. This further reduces the sensitivity for specific assessment of utricular function and thus prediction of tumor origin.

Sensitivity and specificity for determining the nerve of origin, however, depend on tumor size^28^. Since larger tumors affect both, IVN and SVN, differentiation might not be meaningful, but also not required. The present cohort included also larger tumors which was the reason for some tumors not being allocated to one of the specific nerves of origin groups.

The study results are limited by the power and sample size. However, sufficient power was observed for the cVEMP ARs in the IVN group. We conclude that the cVEMP amplitude reduction may be specific for IVN origin. In the patients with SVN tumor origin, however, the AR difference was so small that a sample size of 61 would be required for adequate power. This is very high for such a rare disease and also reduces the power on an individual level.

Since bone conduction vibration is the preferred stimulus for elicit oVEMPs, a powerful transducer with sufficient maximum output force level is required. The used transducer (B250) was applicable to all participants of the clinical cohort. The concave surface of the transducer allowed proper fit to the curved mastoids in most cases while a spring band ensured the required static force. The maximum stimulation level of 150 dB force level induced hearing sensations as well, especially in the non-affected side. As sufficient VEMP responses could be measured at that level, a further level increase does not appear necessary, avoid the risk for noise-induced hearing loss compared to air-conducted stimulation.

We conclude that B250 transducer stimulation is feasible for assessing vestibular function in patients with sporadic VS as it gives clinically viable VEMP responses in more patients than when using AC and B81. It was easy to attach with the steel spring headband and its compatibility with the Eclipse system made it simple to both calibrate and use. Bone-conducted VEMP measurements may be used to determine the nerve of origin in VS with neither the risk for exposing patients to hazardous high sound levels nor absence of responses in patients with conductive hearing loss.

## Data Availability

Raw data may be provided by the corresponding author on request.
